# Assessment of Climate Change and Human Activities on Vegetation Development in Northeast China

**DOI:** 10.3390/s22072509

**Published:** 2022-03-25

**Authors:** Lin Xue, Martin Kappas, Daniel Wyss, Chaoqun Wang, Birgitta Putzenlechner, Nhung Pham Thi, Jiquan Chen

**Affiliations:** 1 Department of Cartography, GIS and Remote Sensing, Institute of Geography, University of Goettingen, 37077 Goettingen, Germany; mkappas@gwdg.de (M.K.); daniel.wyss@uni-goettingen.de (D.W.); birgitta.putzenlechner@uni-goettingen.de (B.P.); 2 Biogeochemistry of Agroecosystems, University of Goettingen, 37077 Goettingen, Germany; chaoqun.wang@forst.uni-goettingen.de; 3 Faculty of Rural Development, Hue University of Agricultural and Forestry, Hue University, Hue 53000, Vietnam; ptnhung@hueuni.edu.vn; 4 Department of Geography, Environment, and Spatial Sciences, Michigan State University, East Lansing, MI 48823, USA; jqchen@msu.edu; 5 Center for Global Change and Earth Observations, Michigan State University, East Lansing, MI 48823, USA

**Keywords:** SPEI, vegetation development, water availability, climate change, human activities, Chinese ecological restoration projects, northeast China

## Abstract

Vegetation in Northeast China (NEC) has faced dual challenges posed by climate change and human activities. However, the factors dominating vegetation development and their contribution remain unclear. In this study, we conducted a comprehensive evaluation of the response of vegetation in different land cover types, climate regions, and time scales to water availability from 1990 to 2018 based on the relationship between normalized difference vegetation index (NDVI) and the standardized precipitation evapotranspiration index (SPEI). The effects of human activities and climate change on vegetation development were quantitatively evaluated using the residual analysis method. We found that the area percentage with positive correlation between NDVI and SPEI increased with time scales. NDVI of grass, sparse vegetation, rain-fed crop, and built-up land as well as sub-humid and semi-arid areas (drylands) correlated positively with SPEI, and the correlations increased with time scales. The negatively correlated area was concentrated in humid areas or areas covered by forests and shrubs. Vegetation water surplus in humid areas weakens with warming, and vegetation water constraints in drylands enhance. Moreover, potential evapotranspiration had an overall negative effect on vegetation, and precipitation was a controlling factor for vegetation development in semi-arid areas. A total of 53% of the total area in NEC showed a trend of improvement, which is mainly attributed to human activities (93%), especially through the implementation of ecological restoration projects in NEC. The relative role of human activities and climate change in vegetation degradation areas were 56% and 44%, respectively. Our findings highlight that the government should more explicitly consider the spatiotemporal heterogeneity of the influence of human activities and water availability on vegetation under changing climate and improve the resilience of regional water resources. The relative proportions and roles map of climate change and human activities in vegetation change areas provide a basis for government to formulate local-based management policies.

## 1. Introduction

Global warming has exerted significant impacts on vegetation, reflected by its composition, distribution, and phenology [1,2,3]. Previous studies have reported that global drylands have expanded under a warming climate [4,5]. By 2100, under a high greenhouse gas emissions scenario, when climate is forecast to be warmer than it is now, almost all land areas will become drier except India and northern tropical Africa, especially for arid areas [4]. Vegetation growth is expected to become more water constrained because global warming results in an increase in vapor pressure deficit and possible reductions in soil moisture [6]. Understanding how vegetation responds to water availability in the context of global warming results in larger concerns [7,8]. However, quantifying vegetation response to water availability at large spatial and temporal scales is challenging as vegetation development response to water availability is influenced by many interacting factors including geomorphology and soils, vegetation types, and time scales [7,9,10]. For example, in arid areas where vegetation is restricted by water deficits, precipitation shows a positive effect on vegetation development [7] while in humid areas subjected to waterlogging or at high latitudes, short-term precipitation deficiency may result in higher temperature and radiation, leading to enhanced vegetation growth [11,12]. 

Northeast China (NEC) has the largest area of freshwater marshes in China. It is a typically sensitive area toward climate change and has been regarded as a major ecological barrier for northern China and even Northeast Asia, playing an important role in protecting biodiversity and regulating the regional climate of Northeast Asia [13,14]. Previous studies have shown that NEC is one of the hot spot warming areas in East Asia [15,16]. Additionally, almost 45% of vegetation in NEC is at moderate or high risk of meteorological drought, which may cause vegetation reduction and ecological environment deterioration [17]. In turn, vegetation changes are commonly considered as indicators of environmental changes at various spatial and temporal scales. Recent studies have documented vegetation response to climate change in NEC in terms of the relationship between temperature, precipitation, and vegetation status [13,18], vegetation drought risk to climate change [17], and the response of different vegetation types to water availability and the timescale of vegetation response to drought [7]. Despite the growing interest in vegetation growth trends in response to changing climate, there is still a lack of comprehensive assessments of long-term vegetation response to water availability using the full satellite record. NEC has rich vegetation resources and diverse climate regions characterized by different hydrothermal conditions. Revealing the response of vegetation in different land cover types and climate regions to water availability at multi-time scales is beneficial to strengthening the understanding of the vegetation response to drought severity and duration.

Human activities are also critical factors affecting vegetation changes. NEC is known for its natural resources, grain production, and industrial base. Since the 1950s, large demands for food and economic development have led to natural vegetation degradation, wetland reduction, and prominent desertification and salinization in NEC [19,20]. Chinese central and local governments have gradually realized the importance of the sustainable utilization of forests in NEC. The *Natural Forest Conservation Project* and the *Grain to Green Project* were implemented in 1998 and 1999 to improve and protect forests [21]. In addition, to restore ecological problems, the *Ecological Function Conservation Project* and the *Three Norths Shelter Forest System Project* were implemented in 2005 and 2011, respectively [22]. Mao et al. (2019) [22] and Li et al. (2021) [18] documented trends in NEC’s greening and productivity growth after the implementation of these projects. However, the resulting increase in vegetation greenness may also lead to excessive depletion of deep soil water through excessive evapotranspiration, thereby limiting vegetation development [23].

Climate change and human activities, individually or collectively, affect vegetation greenness. An in-depth understanding of how climate change and human activities affect vegetation on long-term time scales has important practical significance for the management of human activities and the restoration of vegetation. However, the factors dominating vegetation changes in NEC remain unclear and cannot be precisely discussed. There is still a lack of quantitative assessment of the long-term response of vegetation to climate change and human activities. The residual trends method (RESTREND) [24] allows for the separation of human activities from climate influences on vegetation by developing an NDVI climate model, which yields quantitative results. This method is simple and effective, is generally accepted as a robust approach to reflect spatial drivers well, and is commonly used in water-limited ecosystems [25,26,27].

Here, we evaluated spatiotemporal heterogeneity of the influence of changing water availability on vegetation over the last three decades in NEC. We used normalized difference vegetation index (NDVI) as a proxy of vegetation development and standardized precipitation evapotranspiration index (SPEI) aggregated multi time scales as a proxy of water availability. SPEI normalizes the difference between precipitation (PRE) and potential evapotranspiration (PET) according to a specific distribution and characterizes the water deficit condition [28]. Importantly, SPEI is more sensitive to drought severity caused by the rapid rise in temperature (TEM) [28]. As an indicator of vegetation development, status, and spatial distribution density, NDVI has high sensitivity and spatial and temporal adaptability to detect vegetation compared to other vegetation indexes [29,30]. The statistical relationship between NDVI and multi time scales SPEI has been widely applied to examine the response of vegetation development to water availability [7,8,27]. Moreover, the residual trend method was applied to effectively separate and quantitatively assess the impacts of climate change (e.g., PRE and PET) and human activities on vegetation dynamics. We aimed to explore the characteristics of vegetation facing climate risks and human activities within different land cover types, climate regions, and time scales, which has important practical significance for coping with future climate changes and formulating more detailed strategies of vegetation restoration and management in NEC.

## 2. Materials and Methods

### 2.1. Study Area

**Figure 1:**
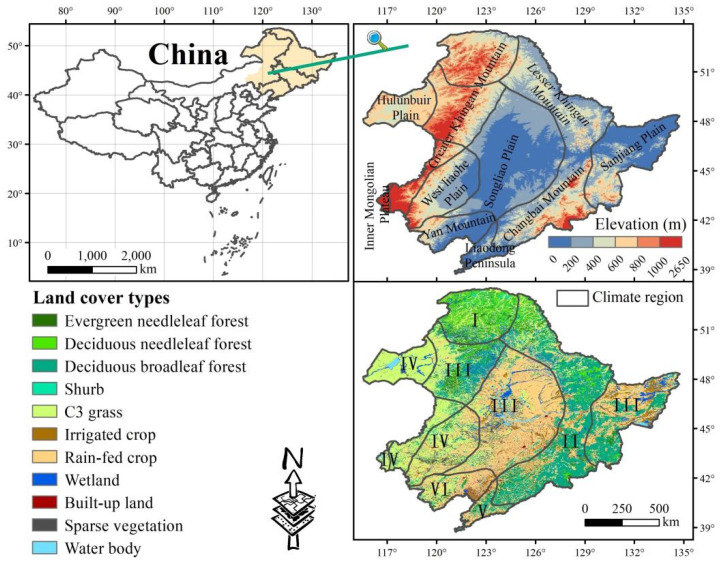
The location, elevation, climate region, and land cover of Northeast China (NEC). The climate regions were based on the climate regionalization in China [31], which includes the cold temperate humid region (C-humid, I), middle temperate humid region (M-humid, II), middle temperate sub-humid region (M-sub humid, III), middle temperate semi-arid region (M-semi arid, IV), warm temperate humid region (W-humid, V), and warm temperate sub-humid region (W-sub humid, VI). The criteria of temperature zones and moisture regions in climate regionalization are shown in Table S1. The elevation data in a 30 m spatial resolution were obtained from the Data Center for Resource and Environmental Sciences of Chinese Academy of Sciences (http://www.resdc.cn/, accessed on 11 March 2021).

NEC region includes northeastern Inner Mongolia, Liaoning, Jilin, and Heilongjiang Provinces (115°32′–135°09′ E, 38°42′–53°35′ N; 0–2650 m a.s.l) and covers an area of approximately 1.24 million km^2^ (Figure 1). It is an important grain-producing and ecological conservation area in China [14]. The topography is characterized by central plains surrounded by mountain ranges. The mountain ranges are mainly composed of Greater Khingan Mountains in the west, Lesser Khingan Mountains in the north, and the Changbai Mountains in the east. Due to the demand for economic development, forest resources in the mountains have been greatly developed and utilized. At low altitude areas that are composed of the Songliao and Sanjiang Plains, human activities are dominated by urbanization and agricultural activities. NEC is dominated by a temperate continental monsoon climate with hot and rainy summers as well as cold and dry winters. The average annual precipitation ranges from 300 to 1000 mm, and the annual average temperature varies between −3 and 10 °C, showing an uneven spatial distribution. From south to north, NEC spans the warm-, middle-, and cold temperate zones while from east to west, NEC broadly transitions from humid- to sub-humid-, and semi-arid areas, accounting for 25%, 46%, and 29% of the total area, respectively. As the area is affected by different hydrothermal conditions, it contains various types of land cover, with the main ones being forest, grass, and rain-fed crops accounting for 29.8%, 23.3%, and 30.9% of the total area, respectively.

### 2.2. Data Sources

Land cover was derived from the “plant functional type map” and the “land cover map (2010)”, land cover types in NEC with eleven types (Figure 1). The “plant functional type map” of China in a 1 km spatial resolution was obtained from the National Tibetan Plateau Data Center. The data have been widely used in ecological research and vegetation dynamic simulation in China by climatologists [32]. Furthermore, we used the “land cover map (2010)” in a 1 km spatial resolution, which was obtained from the Data Center for Resource and Environmental Sciences of Chinese Academy of Sciences (http://www.resdc.cn/, accessed on 11 March 2021) to further subdivide the crops contained in the “plant functional type map” into rain-fed and irrigated crops.

The monthly mean TEM and PRE data in a 1 km spatial resolution from 1990 to 2018 were obtained from the Loess Plateau SubCenter, National Earth System Science Data Center, National Science and Technology Infrastructure of China (http://loess.geodata.cn, accessed on 20 May 2021). These data were spatially downscaled to 1 km from the 30′ Climatic Research Unit dataset with the climatology dataset of WorldClim using the delta spatial downscaling method [33]. These were evaluated using observations collected in 1951–2016 by 496 weather stations across China, and the verification results are credible [33]. The monthly mean PET data in a 1 km spatial resolution were downloaded (http://loess.geodata.cn, accessed on 20 May 2021), which was calculated from air temperature data using the Hargreaves method [34].

The third-generation Global Inventory Modeling and Mapping NDVI dataset (GIMMS3g) were produced by the National Oceanographic and Atmospheric Administration (NOAA) using the Advanced Very High-Resolution Radiometer (AVHRR) instruments (https://climatedataguide.ucar.edu/climate-data/ndvi-normalized-difference-vegetation-index-3rd-generation-nasagfsc-gimms, accessed on 1 June 2021). The dataset provides the longest global NDVI time series from 1981 to the present with a spatial resolution of 8 km and a temporal resolution of 15 days. In this study, we extracted the GIMMS3g data for NEC from 1990 to 1997 and synthesized monthly scaled data using the maximum value composite (MVC) technique [35], which can reduce the interference caused by cloud, aerosol, and solar altitude angles. To match the spatial resolution of the climatic data, the spatial resolution of the GIMMS3g NDVI data were bilinearly resampled to 1 km. In addition, we obtained China’s monthly NDVI dataset in a 1 km spatial resolution from 1998 to 2018, which was generated by SPOT/VEGETATION PROBA-V 1 KM PRODUCTS on the basis of ten days’ maximum value composites (MVC). This was released by the Resources and Environment Science Data Center (RESDC) of the Chinese Academy of Sciences [36] (http://www.resdc.cn/, accessed on 5 June 2021). Compared to GIMMS NDVI data, SPOT/VEGETATION NDVI represents a significant improvement in spatial and spectral resolution, and its NDVI values are more sensitive to changes in vegetation [29,37,38,39]. All datasets were re-projected to a common geographic coordinate reference system (CRS) using GCS Krasovsky_1940.

### 2.3. Data Analysis

SPEI calculated over a specific time scale represents the cumulative water balance over the total months of the specified time period (e.g., SPEI-12 represents the 12-month cumulative water balance) [7,28]. Decreasing SPEI means that conditions become drier, and vice versus. If the SPEI value is less than minus one standard deviation, it is referred to as a drought. In this study, we calculated the SPEI for 1-(SPEI-1), 3-(SPEI-3), 6-(SPEI-6), and 12-month (SPEI-12) periods by using monthly PRE and PET data to reflect the impact of short-term, seasonal, mid-term, and interannual drought variation on vegetation. The complete calculation procedure for the SPEI can be found in Vicente-Serrano et al. (2010) [28].

The non-parametric methods of the Theil–Sen (TS) estimator [40] and Mann–Kendall (MK) test need not meet a specific distribution [41,42,43] and were performed to analyze the magnitude of long-term time series grid trends and to examine whether the trends were significant. The changing trend was divided into the following categories: increase (Z > 0), decrease (Z < 0), significant decrease (Z < −1.96), and significant increase (Z > 1.96). We then analyzed the temporal trends of long-term time series data over the period of 1990–2018. Meanwhile, the 5-year moving average method was applied to smooth out time series fluctuations and highlight the trends, and the linear trend analysis was applied to examine linear trends.

To examine the spatiotemporal relationship between vegetation and water availability, the Pearson correlation analysis method was conducted to calculate the Pearson correlation coefficients between monthly NDVI and multi-temporal time scales of SPEI (SPEIs) during the growing season (April to October) pixel by pixel over NEC from 1990 to 2018 (Equation (1)). Moreover, we extracted the absolute maximum correlation coefficient of NDVI and SPEI for each time scale (Equation (2)). Then, we kept the maximum absolute value of the correlation coefficients between NDVI and SPEIs (RNDVI-SPEIs, Equation (3)) for each pixel and obtained the distribution map of the time scale corresponding to RNDVI-SPEIs (the maximum impact on vegetation). The RNDVI-SPEIs and the corresponding SPEI time scale were effective for modeling the response and resistance of vegetation to drought [10]. The correlation coefficients were calculated as follows:

*R_i,j_* = *cor*(*NDVI_i_*, *SPEI_i,j_*) (1)

*R*_*max*_(*j*) = *max*|*R_i,j_*| (2)

*R_max_* = *max*|*R*_*max*_(*j*)| (3)

where *cor* is the Pearson correlation analysis; *i* represents the *i_th_* month (April to October); *j* is the time scale of SPEI (1-, 3-, 6-, 12- month); and *R_i_*_,*j*_ is the Pearson correlation coefficient. In addition, *R_max_*(*j*) is the absolute maximum correlation coefficient of NDVI and SPEI on the time scale of *j*, and *R_max_* is the absolute maximum correlation coefficient of NDVI and SPEIs.

In addition, the *R_max_*(*j*) and *R_max_* of different land cover types and climate regions were statistically calculated. A positive correlation between NDVI and SPEI indicates that NDVI is increasing with wetting and decreasing with drying, suggesting that vegetation growth is constrained by water scarcity. In contrast, a negative correlation between NDVI and SPEI means that NDVI decreases with wetting and increases with drying, indicating that vegetation growth is constrained by water surplus. In this study, we focused on the maximum water surplus period, which was defined as the maximum time between the start of drying and the last observed positive effect on vegetation development [8].

Furthermore, the contributions of mean PRE and PET to the observed trends of NDVI in the growing season were assessed using partial regression models. The difference between PRE and PET can reflect the water deficit [28], which is the crucial factor for vegetation activity in this region.

Residual analysis method was applied to isolate and quantitatively assess the effects of human activities and climate factors on NDVI dynamics. The method treats the residuals (*NDVI_res_*) between predicted NDVI (*NDVI_pre_*) and observed NDVI (*NDVI_obs_*) as the human influence on vegetation by developing a multiple regression model of NDVI and climate variables [24]. The term *NDVI_pre_*, fitted by a multiple regression model, represents the influence of climatic factors on vegetation. Similar studies have shown that multiple linear regression models can perform well to analyze the relationship between vegetation and climate factors [26,27]. Again, we chose average PRE and PET during the growing season as key climate factors and average NDVI during the growing season as an indicator of vegetation development to establish a multiple regression model. Equations are written as follows:

*NDVI_res_* = *NDVI_obs_* − *NDVI_pre_* (4)

*NDVI_pre_* = *a* × *PRE* + *b* × *PET* + *ε* (5)

where *a* and *b* are the regression coefficients, and *ε* is an error term. 

Similarly, the spatiotemporal trends of *NDVI_pre_* and *NDVI_res_* were detected. An increasing trend in *NDVI_obs_* indicates a positive effect of climate factors or human activities on vegetation development. Taking *NDVI_pre_* as an example, the observed changes in vegetation are only attributed to human activities if the *NDVI_pre_* shows no changing trend over time. An increasing trend of *NDVI_pre_* indicates that climate factors have improved vegetation conditions, and vice versa. The relative role of climate factors and human activities were calculated based on different scenarios among the changing trends of *NDVI_obs_*, *NDVI_res_*, and *NDVI_pre_* (Table 1). All statistical analyses in this study were conducted using R (Version 4.4.0), and image processing was performed in ArcGIS (Version 10.6). The work-flow chart is shown in Figure S1. 

## 3. Results

### 3.1. Spatiotemporal Trending of Climate and NDVI 

**Figure 2:**
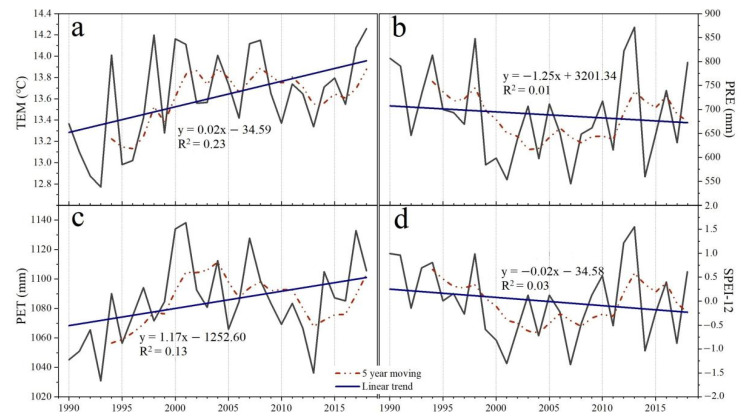
Trends of annual mean temperature (TEM, (**a**)), precipitation (PRE, (**b**)), potential evapotranspiration (PET, (**c**)) during the growing season (April–October), and SPEI-12 (**d**) in Northeast China (NEC) during 1990−2018.

TEM and PET showed increasing trends at rates of 0.02 °C year^−1^ and 1.17 mm year^−1^ over three decades, respectively (Figure 2a,c). There were decreasing patterns for PRE and SPEI-12 at rates of −1.25 mm year^−1^ and −0.02 year^−1^, respectively (Figure 2b,d). The 5-year moving average analysis of TEM indicated an increasing trend from 1990–2008 and 2013–2018 (Figure 2a). PET showed an increasing trend from 1990–2001 and 2013–2018, while reversed patterns were detected for PRE and SPEI-12 (Figure 2b–d).

**Figure 3:**
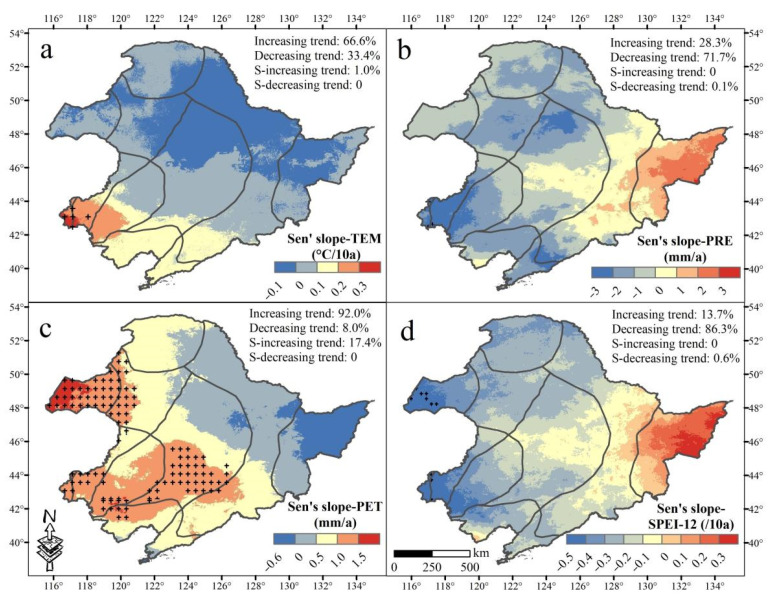
Spatial trends of mean annual temperature (TEM, (**a**)), precipitation (PRE, (**b**)), potential evapotranspiration (PET, (**c**)), and SPEI-12 (**d**) in Northeast China (NEC) during 1990–2018. S- in the figure is the abbreviation for significant. Black crosses indicate significant trends with *p* < 0.05.

An increasing trend in TEM and PET was observed in the past three decades at 66.6% and 92.0% of the total area, respectively (Figure 3a,c). Approximately 28.3% and 13.7% of the total area exhibited an increasing trend for PRE and SPEI-12, respectively. These areas were mainly distributed in the Sanjiang Plain and its southern mountains (Figure 3b,d). Moreover, PRE and SPEI-12 showed decreasing trends from east to west, whilst PET showed increasing trends (Figure 3b–d). It is worth noting that 17.4% of the area showed a significant increasing trend in PET, especially in the west of NEC (Figure 3c).

**Figure 4:**
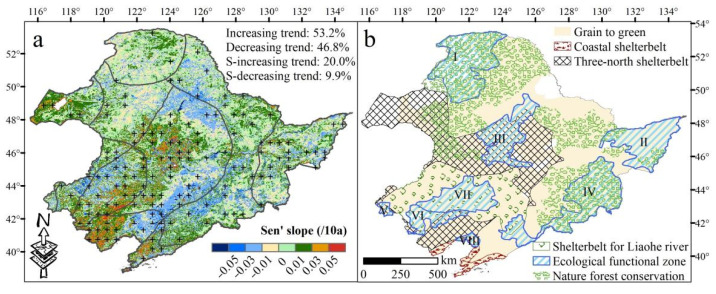
Spatial changes in trending of observed NDVI (*NDVI_obs_*, (**a**)), and spatial distribution of ecological restoration projects (**b**) in Northeast China (NEC). S- in (**a**) is the abbreviation of significant. Black crosses indicate significant trends with *p* < 0.05. The ecological functional zone in (**b**) includes *Grater Khingan Mountain-*(I), *Sanjiang Plain Wetland*-(II), *Songnen Plain Wetland-*(III), *Changbai Mountain-*(IV), Hunshandake Sandy Land-(V), *West Liaohe-*(VI), *Horqin Sandy Land-*(VII), *Liaohe River delta wetland-*(VIII) Ecological Function zone.

The ecological restoration projects in NEC covered almost the entire NEC area (Figure 4b). It can be seen that 53.2% of the total area demonstrated an increasing trend in NDVI (Figure 4a) and 20.0% increased significantly, mainly distributed in the areas where the *Three North-* and *Coastal-Shelterbelt Program*, *Songnen Plain Wetland-*, *Horqin Sandy Land-*, and *West Liaohe-Ecological Function zone* have been implemented (Figure 4a,b). While 9.9% of the total area showed a significant decrease in NDVI, mainly located in the transition zone between the mountains and plains (Figure 4a). Moreover, the temporal trends of *NDVI_obs_* showed an increasing trend from 2001 to 2018 and a decreasing trend from 1990 to 2001 (Figure S2). The area percentage of increasing NDVI trends was higher in the land cover classes: shrubs (63.4%), forests (56.0–62.1%), grass (58.8%), and sparse vegetation (56.3) (Table S2). The area percentage of decreasing NDVI trends was higher in the land cover classes: built-up land (65.1), irrigated crop (62.7%), wetland (56.0%), and rain-fed crops (53.8%), respectively (Table S2). Significant increase or decrease in NDVI was more pronounced for sparse vegetation (30.2%) or built-up land (29.9%), respectively (Table S2). Moreover, deciduous needleleaf forest showed the lowest area percentage of significant shift (Table S2). 

### 3.2. The Spatiotemporal Relationship between NDVI and Climate Factors 

The area percentage with positive correlation between NDVI and SPEI increases with time scales (Figure S3). The relationship between NDVI and SPEI-12 was mainly positive (86.2%) (Figure S3d). Specifically, the NDVI of grasses, sparse vegetation, rain-fed crops, and built-up land as well as sub-humid and semi-arid areas (drylands) positively correlated with SPEI, and increased with time scales (Figure S4) while the NDVI of forests, shrubs, wetlands as well as humid areas was negatively correlated with SPEI-1, SPEI-3, and SPEI-6 (Figure S4). Irrigated crops negatively correlated with SPEI-1 (Figure S4a). This illustrates that the maximum water surplus period (maximum time between start of drying and the last observed positive effect on vegetation development) for irrigated crops and forests, shrubs, wetlands, humid areas were 1-month and 6-months, respectively. 

**Figure 5:**
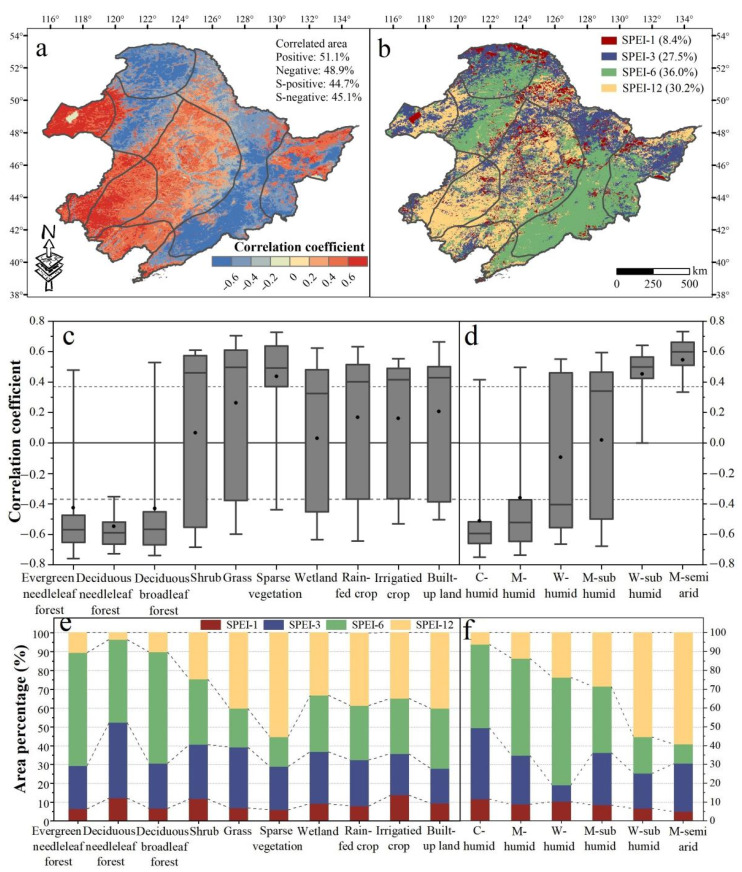
Spatial distribution of the maximum absolute value of correlation coefficients between NDVI and multi time scales SPEI (RNDVI-SPEIs) during the growing season (April–October) over the last three decades (**a**), the spatial distribution of the corresponding time scale of RNDVI-SPEIs during the growing season over the last three decades (**b**), the statistical distributions of RNDVI-SPEI for different land cover types (**c**) and climate regions (**d**), and the area percentage occupied by each time scale corresponding to RNDVI-SPEIs for different land cover types (**e**) and climate regions (**f**). S-in (**a**) is the abbreviation for significant. The maximum and minimum extents of the grey boxes in (**c**,**d**) indicate the 25th and 75th percentiles, the line and dot in each box indicate the median and mean, the whiskers represent the 5th and 95th percentiles, respectively, and the dotted lines indicate significant Pearson correlations with *p* < 0.05.

Furthermore, the maximum absolute value of correlation coefficients between NDVI and multi-time scales SPEI (RNDVI-SPEIs) and the distribution of the time scale corresponding to RNDVI-SPEIs (the maximum impact on vegetation) were extracted (Figure 5). Areas with positive RNDVI-SPEIs accounted for 51.5% of total area, mainly located in drylands, while the negatively correlated area was concentrated in humid areas (Figure 5a,d). The vegetation was most vulnerable to SPEI-6 (36.0%), as can mainly be observed in the Greater Khingan- and Changbai Mountains, followed by SPEI-12 (30.2%) and SPEI-3 (27.5%), and was least sensitive to SPEI-1 (8.4%) (Figure 5b). Specifically, forests and shrubs as well as humid areas were sensitive to SPEI-6 (Figure 5e,f). Sparse vegetation (55%) as well as the middle temperate semi-arid region (M-semi arid, 59%) and warm temperate sub-humid region (W-sub humid, 55%) were most vulnerable to SPEI-12.

The RNDVI-SPEIs varied according to land cover types and climate regions. Significant negative RNDVI-SPEIs were detected for forests and were most pronounced in deciduous needleleaf forests (−0.55) (Figure 5c). The positive RNDVI-SPEIs were found for other land cover types of which sparse vegetation (0.44) showed a significant positive correlation, followed by grassland (0.26), built-up land (0.21), crops (0.17), shrubs (0.07), and wetlands (0.03) (Figure 5c). Moreover, significantly positive and negative RNDVI-SPEIs were observed for M-semi arid (0.54), W-sub humid (0.45), and the cold temperate humid region (C-humid, −0.51), respectively (Figure 5d). Remarkably, RNDVI-SPEIs in humid areas gradually decreased with warming, while RNDVI-SPEIs in drylands gradually increased (Figure 5d and Figure S4b). This means that vegetation water surplus in humid areas weakens with warming, and vegetation water constraints in drylands enhance.

**Figure 6:**
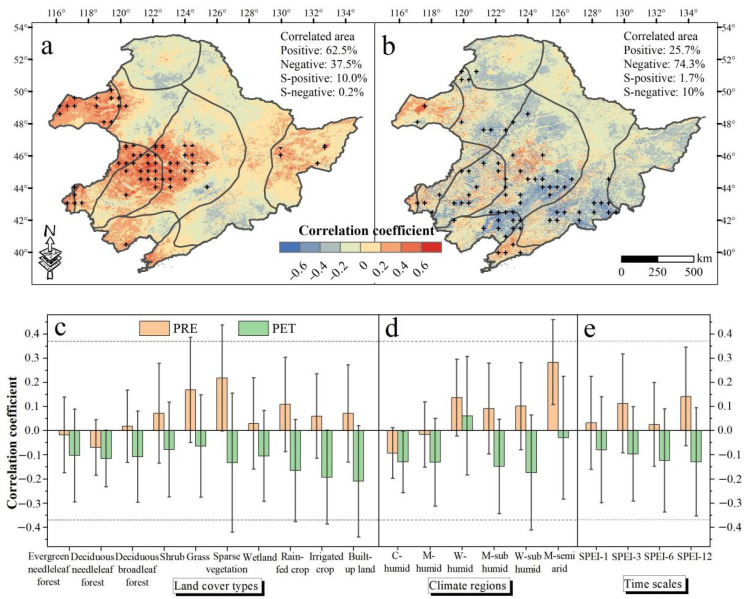
Spatial distribution of the partial correlations between precipitation (PRE) and NDVI (**a**), and between potential evapotranspiration (PET) and NDVI (**b**); the statistical distributions of the partial correlations for different land cover types (**c**), climate regions (**d**), and time scales (**e**) (i.e., the area corresponding to the time scale with the largest absolute value of the correlation coefficient between NDVI and multi time scales SPEI. S- in (**a**,**b**) are the abbreviation for significant. Black crosses indicate significant partial correlations with *p* < 0.05. The data in (**c**–**e**) are the means ± standard deviations. The dotted lines in (**c**–**e**) indicates significant Pearson correlations with *p* < 0.05.

TEM and PET appeared to negatively affect NDVI, with NDVI and PET significantly correlated at *p* < 0.05 (Figure S5a,c). In contrast, a significantly positive relationship between PRE and NDVI was observed (Figure S5b). In addition, the spatial distribution of the partial correlations between NDVI and climate factors indicated that 62.5% and 25.7% of the total area exhibited positive partial correlations between PRE and NDVI and between PET and NDVI, respectively (Figure 6a,b). Specifically, the average partial correlations between NDVI and PRE were positive for different land cover types except for needleleaf forest and were most pronounced for sparse vegetation (0.22) and grass (0.17), and least for wetland (0.03) (Figure 6c). The negative average partial correlations between NDVI and PET were observed for all land cover types. Moreover, the NDVI of built-up land (−0.21), irrigated- (−0.19), and rain-fed crops (−0.21) were most susceptible to PET (Figure 6c). NDVI of the M-semi arid region was most or least sensitive to PRE or PET, respectively. With the extension of time scales, the absolute value of partial correlation coefficients between NDVI and PRE and between NDVI and PET showed an overall increasing trend (Figure 6e).

### 3.3. The Contributions of Climate and Human Activities to NDVI

**Figure 7:**
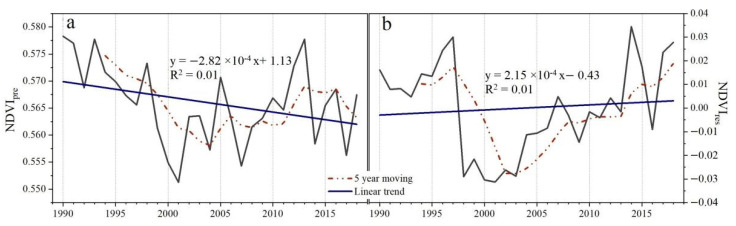
The temporal trends of NDVI impacted by climate factors (*NDVI_pre_*, (**a**)) and NDVI impacted by human activities (*NDVI_res_*, (**b**)) in Northeast China (NEC).

We found that *NDVI_pre_* (NDVI impacted by climate change) mainly showed a decreasing trend (73.5%), while *NDVI_res_* (NDVI impacted by human activities) mainly displayed an increasing trend (60.3%) in NEC over the last three decades. In addition, the *NDVI_pre_* or *NDVI_res_* showed decreasing or increasing trends at a rate of −2.82 × 10^−4^ year^−1^ or 2.15 × 10^−4^ year^−1^ over three decades, respectively (Figure 7a,b). Specifically, decreasing trends from 1990 to 2001 and 2013 to 2018 and an increasing trend from 2001 to 2013 were observed in *NDVI_pre_* (Figure 7a). The temporal trends of *NDVI_res_* showed an increasing trend from 2001 to 2018 and a decreasing trend from 1990 to 2001 (Figure 7b). 

The relative proportions and roles map of climate change and human activities on vegetation development in NEC is shown in Figure S7. Overall, human activities contributed much more to vegetation dynamics than climate change, especially in the areas with an increase in NDVI. The contribution of climate change accounted for 24.5% of total variation in vegetation, and the rate was 7.3% in the area with increased NDVI and 44.4% in the area with decreased NDVI (Figure S7a,c,e). A total of 75.5% of the total variation in vegetation was explained by human activities with 92.7% in the area with increased NDVI and 55.6% in the area with decreased NDVI (Figure S7b,d,f).

**Figure 8:**
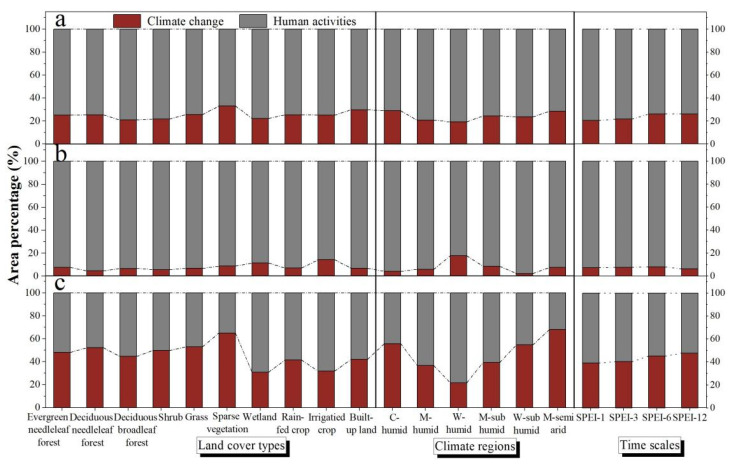
The relative proportions and roles of climate change and human activities on areas with observed NDVI (*NDVI_obs_*) changes (**a**), *NDVI_obs_* increasing (**b**), and *NDVI_obs_* decreasing (**c**) in different land cover types and time scales (i.e., the area corresponding to the time scale with the largest absolute value of the correlation coefficient between NDVI and multi time scales SPEI.

In the area with increased NDVI, human activities were the predominant roles in all land cover types. The contribution of climate change was small, being more pronounced for wetlands (11.4%) and irrigated crops (14.3%) (Figure 8b). In contrast, in areas where NDVI decreased, the relative role rate of human activities to vegetation change was still significant, ranging from 35% (sparse vegetation) to 69% (wetland), while the effects of climate change were more pronounced for forests (44.6–52.2%), shrubs (49.8%), grass (53.2%), and sparse vegetation (65.0%) (Figure 8c). For climate regions, the effects of climate change were more pronounced in the vegetation degraded areas of M-semi arid (72%). The relative role rate of climate change increased as the time scale lengthened, whereas human activities showed an opposite pattern (Figure 8a–c).

## 4. Discussion

### 4.1. Vegetation Response to Water Availability and Its Controlling Factors

Reduced water availability may have positive or negative effects on vegetation development. Our findings demonstrate that most of the areas with positive RNDVI-SPEIs were located in drylands or areas mainly covered by grass, sparse vegetation, rain-fed crops, and built-up land. This indicates that vegetation development in these areas was constrained by water scarcity. These results are consistent with expectations that most drylands experience water constraints [44]. In contrast, most negative RNDVI-SPEIs were found in areas located in humid areas or areas dominated by forests, shrubs, irrigated crops, and wetlands. These areas were mainly distributed in the Greater- and Lesser- Khingan Mountains and Changbai Mountain, with the highest elevations within NEC. This suggests that vegetation was degraded under wetter conditions and more greening occurred under drier than normal conditions, which is supported by the results of studies in other humid areas [12,45]. The temperature and solar radiation are limiting factors in humid areas or in areas with high latitudes and altitudes [46]. Thus, short-term precipitation deficiency may result in higher temperatures, leading to enhanced vegetation development [12,45].

The correlation between NDVI and multi-time scales SPEI and the distribution of the time scale corresponding RNDVI-SPEIs provide information about the response and resistance of vegetation to water deficits [7,10]. A time lag typically exists between the onset of water scarcity and the emergence of observable consequences on vegetation [47,48]. In humid areas or areas covered by forests and shrubs, which experience water surplus, vegetation was most sensitive to SPEI-3 and SPEI-6 (Figure 5e,f). This indicates that the 3- and 6-month water deficits in these areas have the most significant effect on the promotion of vegetation development. However, once the time scale of the water deficit exceeds the maximum water surplus period, vegetation development will be restricted (Figure S4). In contrast, most vegetation in drylands quickly responded to changing water availability (Figure S4). This is shown through the rapid response of sparse vegetation and vegetation in built-up land to SPEI-1 or grass and crops to SPEI-3 (Figure S4a) and is related to the special physiological and functional strategies of vegetation in drylands [7]. Plants respond immediately to water scarcity and reduce water loss, respiration consumption, and photosynthetic activity to adapt to water deficit [49]. Moreover, the positive correlation between NDVI and SPEI in drylands or areas covered by grass, sparse vegetation, crops, and built-up land increased with time scales (SPEI-12 performed better, Figure S4). Sparse vegetation or dryland plants were most vulnerable to SPEI-12 (Figure 5e,f), indicating that the vegetation in such ecosystems has the ability to withstand water scarcity [7]. 

The response of vegetation in different climate regions to water availability was expected to reflect the response trends of vegetation to water availability with warming. Our results indicate that vegetation water surplus in humid areas weakens with warming (Figure 5d and Figure S4b). The vegetation development was limited by TEM or radiation in cold and humid regions [11]. Increased PRE, accompanied by an increase in cloud cover, reduces incident radiation. Reduced incoming solar radiation is not conducive to photosynthesis, thus limiting vegetation development [50]. The rising TEM will neutralize the negative effect of PRE and promote the growth of vegetation. In contrast, vegetation water constraints in drylands enhance with warming (Figure 5d and Figure S4b). Sidor et al. (2015) [51] reported that rising TEM will accelerate vegetation evapotranspiration and reduce soil moisture. In this regard, PRE showed an overall positive effect on vegetation except for most vegetation in humid areas or areas covered by forest (Figure 6c,d). NDVI of the M-semi arid region was most or least sensitive to PRE or PET, respectively, which indicates PRE is the controlling factor for vegetation development in semi-arid areas in NEC while PET had an overall negative effect on vegetation, especially for NDVI of built-up land and crops (Figure 6c). The increase in PET will reduce the available soil moisture and then limit vegetation development [52]. However, with the extension of time scales, the absolute value of partial correlation between NDVI and PRE, and between NDVI and PET showed an increasing trend (Figure 6e), which indicates that the effect of climate factors on vegetation had a time lag. 

### 4.2. Effects of Human Activities on Vegetation Development

The results of residual trend analysis showed that human activities exhibited the dominant effect (93%) in areas where the vegetation status (i.e., NDVI) had improved (Figure 8b and Figure S7d). This is mainly attributed to the implementation of multiple ecological restoration projects in NEC since 1998, as evidenced by the trend of *NDVI_res_*, which increased after 2001 (Figure 7b). In contrast, the temporal trends of NDVIres showed a decreasing trend from 1990 to 2001 (Figure 7b), which means the vegetation affected by human activities has a tendency to degrade from 1990 to 2001. To respond to large amounts of deforestation and associated ecological issues, the *Natural Forest Conservation Project* and the *Grain to Green project* were implemented in NEC in 1998 and 1999, respectively. After the implementation of these two projects, the commercial logging in natural forests in NEC stopped, and large areas of afforestation were observed (Figure 4a,b). Increasing trends were observed in evergreen needleleaf forest, deciduous needleleaf forest, and deciduous broadleaf forest, accounting for 56.5%, 56.0%, and 62.1% of the total area in each type, respectively (Table S2). Mao et al. (2019) [22] revealed that with the implementation of the *Grain to Green project*, 4140 km^2^ of cropland were converted to woodland during 2000–2015 in NEC. Moreover, to reduce water loss, soil erosion, and desertification, the *Ecological Function Conservation Project* was implemented in 2005, and the *Three Norths Shelter Forest System Project* in 2011. We found that the vegetated area significantly improved, mainly distributed in areas where the *Three North-* and *Coastal-Shelterbelt Program*, *Songnen Plain Wetland-*, *Horqin Sandy Land-*, and *West Liaohe-ecological function zone* were implemented, containing measures to conserve and rehabilitate vegetation, wetlands and strengthen sandstorm prevention activities. The area percentage of increasing NDVI trends were higher in forests, shrubs, grass, and sparse vegetation (Figure 4a,b, Table S2). Zhang et al. (2012) [53] reported that the ecological restoration greatly contributed to the increased vegetation cover and prevented sand expansion in the Horqin Sandy Land. Mao et al. (2019) [22] also confirmed that better protection and restoration of the natural ecosystems were achieved in the western part of the NEC including substantial enhancement of sandstorm prevention, habitat provision, and grain production. Enhancing vegetation cover not only increases soil organic carbon and total nitrogen, but also improves ecosystem services, reduces soil and water erosion, and improves regional climate [54,55]. Our results proved that the improvement of vegetation under the influence of human activities was accompanied by the overall improvement of climatic conditions and soil moisture conditions in NEC, as evidenced by PRE, and SPEI-12 experienced an increasing trend from 2001 to 2013, while PET showed an opposite trend (Figure 2).

In areas where NDVI decreased, the relative role rate of human activities to vegetation in different land cover types was still obvious, ranging from 35% to 69% (Figure 8c). For example, the effects of human activities for grass degradation accounted for 47% and were mainly located in West Liaohe Plain, located in the middle of Tongliao City of the Inner Mongolia Autonomous Region. A government investigation demonstrated that the total amount of livestock in Tongliao increased significantly since 1990 (http://tj.nmg.gov.cn, accessed on 15 September 2021). These higher stocking numbers led to increasing consumption of grass biomass, accelerating grassland degradation, and reduced grassland resilience [56]. Therefore, the livestock area must control the number of livestock and the time of grazing to promote the restoration of the grassland. The area where forests and shrubs decreased can be attributed to 48–55% human activity. On one hand, this may be related to the occurrence of forest fires during the study period. Studies have shown that forest fires are closely related to human activities, and the fire sites are close to settlements and roads [57]. On the other hand, although the implementation of the ecological project has restored part of the forests cut down in pursuit of economic benefits, the proportion of young and middle-aged forests is large. Some researchers even believe that excessive emphasis on afforestation and ignorance of regional climate, hydrology, and soil conditions may lead to soil degradation, soil moisture reduction, and decreasing survival rates of planted trees [58]. Additionally, the growth of native vegetation will be affected [59]. Mao et al. (2019) [22] reported that the services of water yield have been markedly weakened in NEC, which can be attributed to the substantial enhancement of evapotranspiration induced by increased vegetation cover. Therefore, the government should more explicitly consider the influence of ecological restoration projects on the local soil ecosystem and vegetation and the resilience of regional water resources. The vegetation should be maintained but not further expanded in areas with limited water resources [60]. Apart from this, forest ecosystem quality should be improved to mitigate limited or inappropriate species during afforestation [58]. 

Additionally, our results showed that vegetation degradation in wetland, crops, and built-up land is mainly affected by human activities (Figure 8c). Rapid urbanization and crop expansion are reducing the growth space of vegetation, resulting in the degradation of vegetation around cities (Figure 4a). Peri-urbanized areas are seen as priority areas to improve the coupling of urban development and vegetation restoration [61]. Although extensive areas of cropland have been reconverted into natural land cover after the implementation of multi ecological restoration projects, agricultural cultivation is still the dominant force driving the loss of forests, grass, and wetlands [22]. Furthermore, policy-driven conversion from rain-fed crops to irrigated crops also caused water shortages [62], which further led to loss and degradation of wetlands and grasslands.

Land cover changes further induced ecosystem service degradation in NEC including decreases in water yield, soil retention, and carbon storage, which greatly affected natural vegetation and agricultural production [21,22]. Spatially, the main effect of human activities on vegetation degradation can be seen in the Sanjiang Plain, Changbai Mountain, the Lesser Khingan Mountain, and the northern areas of Songliao Plain (Figure S7f). In addition to losses of forest, grass, and wetland, habitat fragmentation and human disturbances were the most important driving forces in these areas [63]. Priority should be given to the formulation of future human activities management policies in these areas including the restoration of wetlands and proper management of agricultural production and urbanization activities. 

### 4.3. Effects of Climate Change on Vegetation Development

The contribution of climate change was low in areas where NDVI increased, but more pronounced for wetland and irrigated crops (Figure 8b). Short-term water deficit has promoted the growth of vegetation in wetlands and irrigated crops (Figure S4a), possibly resulting in a weakening of wetland ecological functions [45]. In contrast, the effects of climate change on vegetation degradation were especially high for forests (45–52%), shrubs (50%), grass (53%), and sparse vegetation (65%), or in M-semi arid areas (72%). Grass and sparse vegetation are mainly located in drylands such as sandy and saline-alkali land, with severely restricted water. In this study, we proved that the NEC experienced a warming and drying trend from 1990 to 2001 and 2013 to 2018 with vegetation water constraints tending to enhance, as evidenced by the trend in NDVIpre, which decreased from 1990 to 2001 and 2013 to 2018 (Figure 7b). In addition, although short-term, seasonal, and mid-term water deficits promote the growth of forests and shrubs, annual water deficit limits their growth (Figure S4a). Furthermore, the relative role rate of climate change on vegetation change increased with time scale (Figure 8a–c), whilst human activities showed an opposite pattern. This result indicates that human activities tend to have an immediate impact on vegetation, while the impact of climate change on vegetation usually has a time lag and is cumulative. 

## 5. Conclusions

In the present study, most of the areas with positive RNDVI-SPEIs were located in drylands or areas mainly covered by grass, sparse vegetation, rain-fed crops, and built-up land, and the correlations increased with time scales. In contrast, most negative RNDVI-SPEIs were found in areas located in humid areas or areas dominated by forests, shrubs, irrigated crops, and wetland, and vegetation was most sensitive to SPEI-3 and SPEI-6. In addition, we found that vegetation water surplus in humid areas weakens with warming, and vegetation water constraints in drylands enhance. From 1990 to 2018, 53.2% of the total area demonstrated an increasing trend in NDVI, and 20.0% increased significantly, while 9.9% showed a significant decrease trend in NDVI. The results of residual trend analysis demonstrated that human activities exhibited a dominant effect in areas where the vegetation status improved. This was mainly attributed to the implementation of multiple ecological restoration projects in the NEC since 1998. Nevertheless, negative effects of human activities on vegetation development are still obvious due to overgrazing, urbanization, crop expansion, and policy-driven decline in water yield. In the context of a warming and drying climate, climate change contributes strongly to the degradation of vegetation. Furthermore, the direct impact of human activities and the time lag and cumulative effects of climate change on vegetation development were confirmed in this study. Our findings highlight that the government should more explicitly consider the spatiotemporal heterogeneity of the influence of human activities and water availability on vegetation under a changing climate, and improve the resilience of regional water resources. In areas with limited water resources, vegetation should be maintained, but should not be further expanded.

## Figures and Tables

**Table 1:** Methods for assessing the relative proportions and roles of climate change and human activities on vegetation development.

*NDVI_obs_*	*NDVI_pre_*	*NDVI_res_*	Relative Contribution (%)		Description
	*Slope_pre_*	*Slope_res_*	Climate Change	Human Activities	
Increased (Slope_obs_ > 0)	>0	>0	$\frac{Slope_{pre}}{Slope_{obs}}$	$\frac{Slope_{res}}{Slope_{obs}}$	Both climate change and human activities improved the vegetation condition
	>0	<0	100	0	Climate change improved the vegetation condition
	<0	>0	0	100	Human activities improved the vegetation condition
Decreased (Slope_obs_ < 0)	<0	<0	$\frac{Slope_{pre}}{Slope_{obs}}$	$\frac{Slope_{res}}{Slope_{obs}}$	Both climate change and human activities induced the degradation of vegetation
	<0	>0	100	0	Climate change induced the degradation of vegetation
	>0	<0	0	100	Human activities induced the degradation of vegetation

## Data Availability

The climate dataset used for this research is publicly available at http://loess.geodata.cn, accessed on 20 May 2021; the GIMMS3g NDVI data used for this research are publicly available at https://climatedataguide.ucar.edu/climate-data/ndvi-normalized-difference-vegetation-index-3rd-generation-nasagfsc-gimms (accessed on 1 June 2021); and China’s monthly NDVI dataset and land cover map (2010) used for this research are publicly available at http://www.resdc.cn/ (accessed on 5 June 2021).

## Supplementary Materials

The following supporting information can be downloaded at: https://www.mdpi.com/article/10.3390/s22072509/s1, Figure S1: Study work-flow chart; Figure S2: Temporal trends of observed NDVI (*NDVI_obs_*) in Northeast China (NEC); Figure S3: Spatial distribution of the correlation coefficients between NDVI and multi time scales SPEIs during the growing season over the last three decades in Northeast China (NEC); Figure S4: The statistical distributions of correlation coefficients between NDVI and multi time scale SPEI for different land cover types (a) and climate regions (b), respectively; Figure S5: Linear relationships between NDVI and temperature (TEM, a), precipitation (PRE, b), and potential evapotranspiration (PET, c) in Northeast China (NEC); Figure S6: Spatial distribution of the trends of NDVI impacted by climate change (*NDVI_pre_*, a) and NDVI impacted by human activities (*NDVI_res_*, b); Figure S7: The spatial distribution of the relative proportions and roles of climate change and human activities in areas with observed NDVI (*NDVI_obs_*) changes (a,b), *NDVI_obs_* increasing (c,d), and *NDVI_obs_* decreasing (e,f), respectively; Table S1: Criteria of temperature zones and moisture regions in climate regionalization; Table S2: Area percentage of the NDVI trends of different land cover types.
