# Supplementary material for: Assessment of Climate Change and Human Activities on Vegetation Development in Northeast China
Source: Sensors (Basel). 2022 Mar 25;22(7):2509. doi: 10.3390/s22072509 (PMC9002475; doi:10.3390/s22072509)
Supplement: Supplementary file 1 [file sensors-22-02509-s001.zip › sensors-1630829-supplementary.pdf]

Supplementary Materials: Assessment of Climate Change and Human Activities on Vegetation Development in Northeast China

Lin Xue, Martin Kappas, Daniel Wyss, Chaoqun Wang, Birgitta Putzenlechner, Nhung Pham Thi and Jiquan Chen

Table S1. Criteria of temperature zones and moisture regions in climate regionalization.

Temperate Zone	Main Indicators (Days with Average Daily Temperature ≥ 10℃ (d))	Moisture Region	Main Indicators (Dryness Index)
Cold temperate	< 100	Humid	≤ 1.00
Middle temperate	100 ~ 170	Sub-humid	1.00 ~ 1.50
Warm temperate	170 ~ 220	Semi-arid	1.50 ~ 4.00

Table S2. Area percentage of the NDVI trends of different land cover types.

Land Cover Types	Area Percentage NDVI Trends (%)			
	**Increasing**	**Decreasing**	**S-Increasing**	**S-Decreasing**
Evergreen needleleaf forest	56.5	43.5	18.1	4.6
Deciduous needleleaf forest	56.0	44.0	5.7	1.4
Deciduous broadleaf forest	62.1	37.9	21.2	3.8
Shrub	63.4	36.6	24.8	7.4
Grass	58.8	41.2	21.6	8.2
Sparse vegetation	56.3	43.7	30.2	17.1
Wetland	44.0	56.0	16.5	11.2
Rain-fed crop	46.2	53.8	20.8	14.0
Irrigated crop	37.3	62.7	14.8	17.4
Built-up land	34.9	65.1	18.2	29.9

S- in the table is the abbreviation for significant.

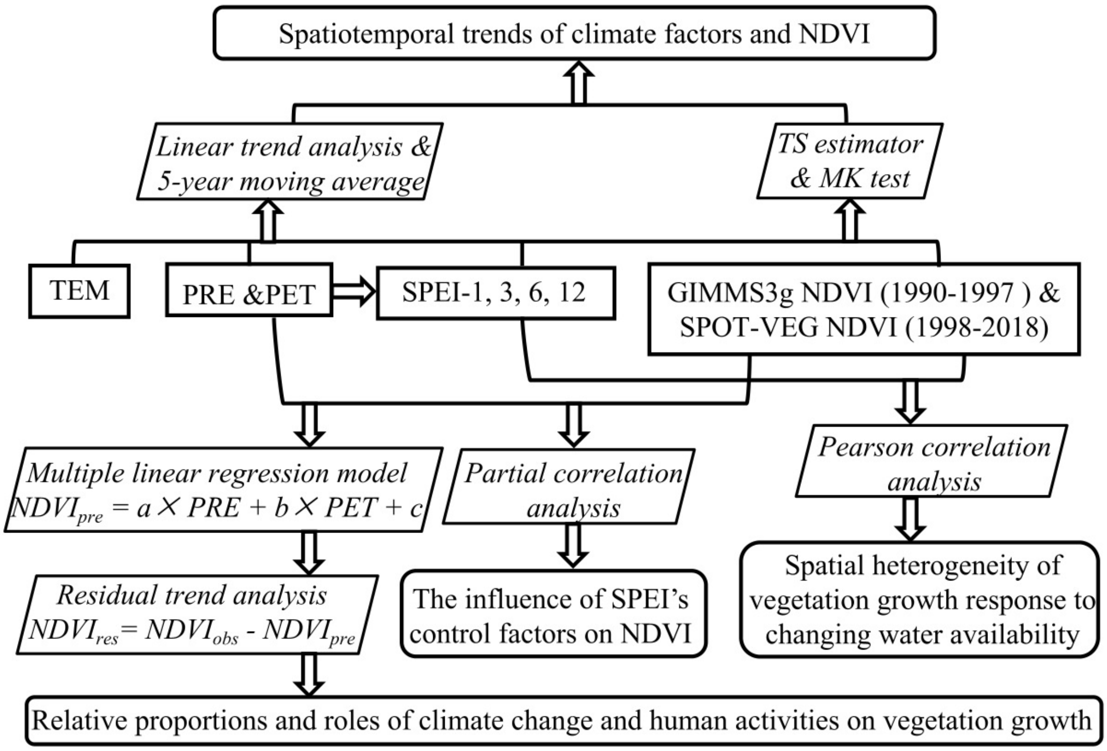

Figure S1. Study work-flow chart

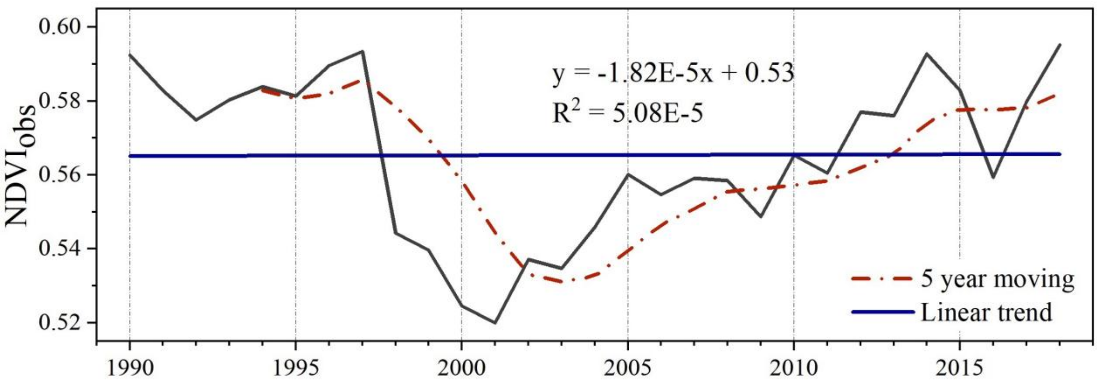

Figure S2. Temporal trends of observed NDVI (*NDVI$_{obs}$*) in Northeast China (NEC)

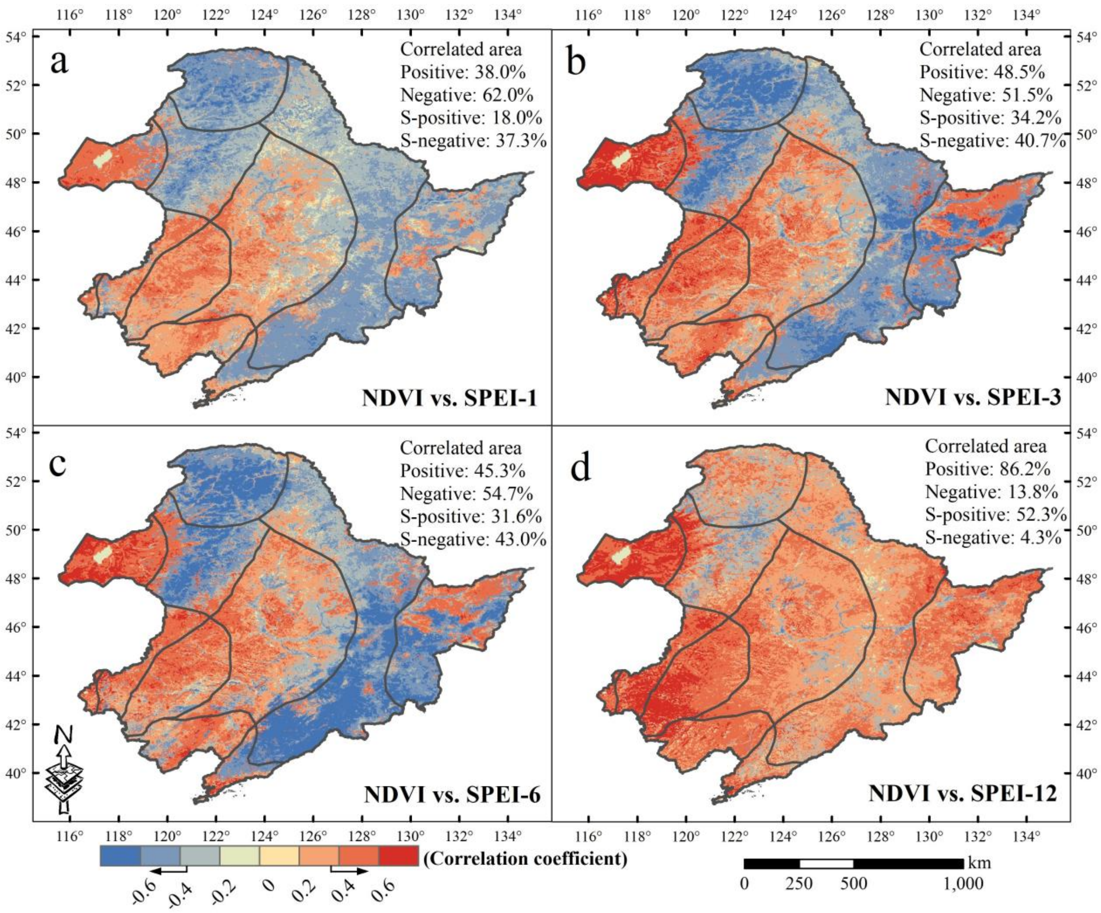

Figure S3. Spatial distribution of the correlation coefficients between NDVI and multi time scales SPEI during the growing season over the last three decades in Northeast China (NEC). The arrow in the legend means that the Pearson correlation coefficients between NDVI and SPEI are significant.

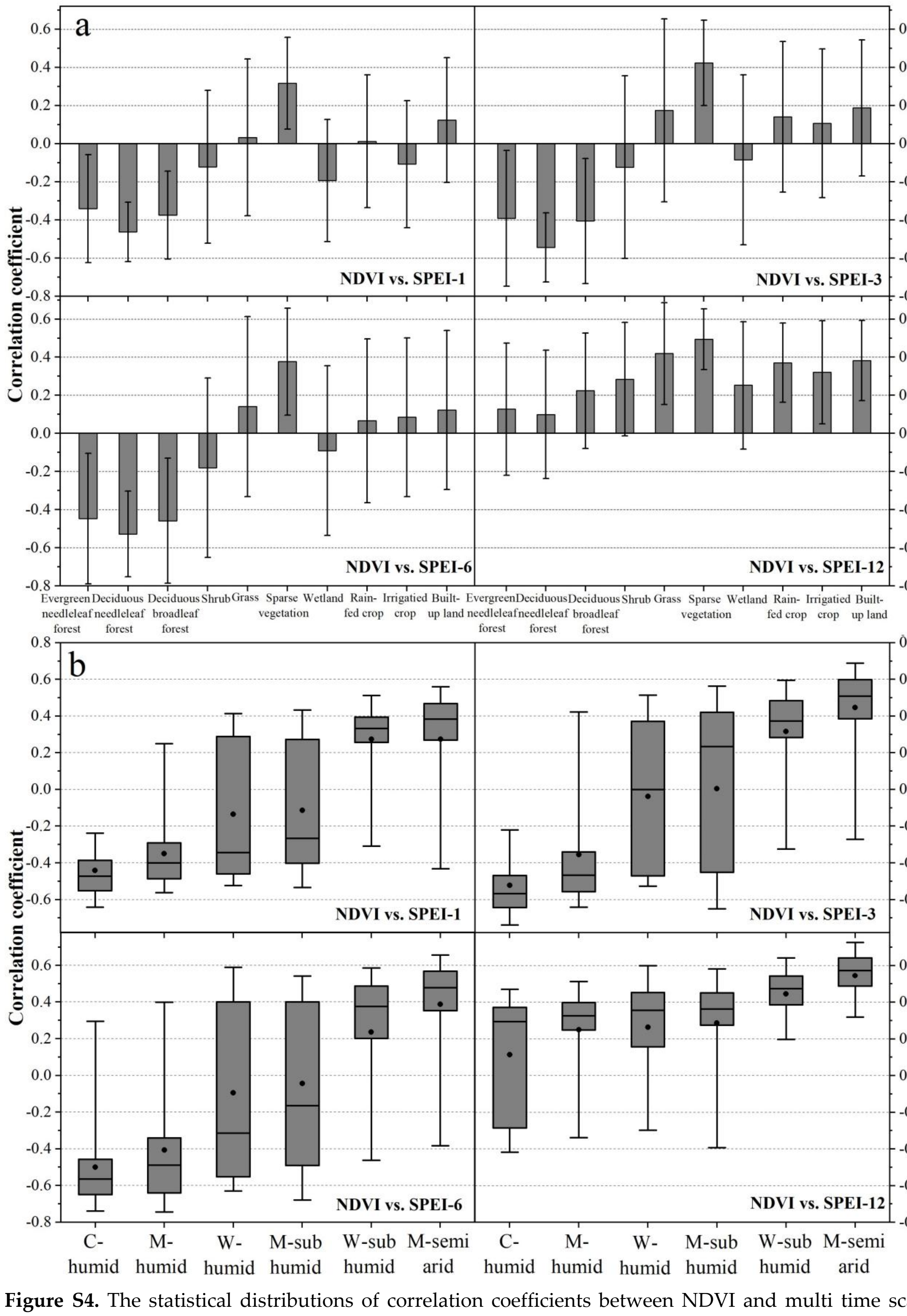

Figure S4. The statistical distributions of correlation coefficients between NDVI and multi time scale SPEI for different land cover types (**a**) and climate regions (**b**), respectively. The data in subfigure a are the means ± standard deviations. The maximum and minimum extents of the grey boxes in subfigure b indicate the 25th and 75th percentiles, the line and dot in each box indicate the median and mean, the whiskers represent the 5th and 95th percentiles, respectively.

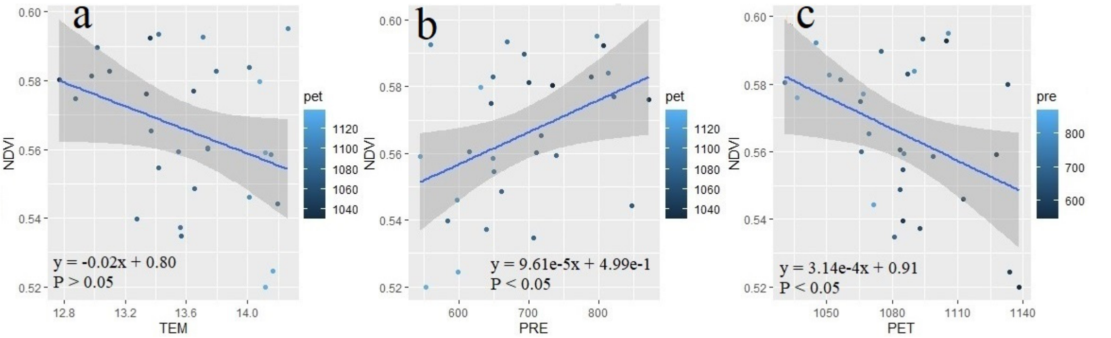

Figure S5. Linear relationships between NDVI and temperature (TEM, **a**), precipitation (PRE, **b**), and potential evapotranspiration (PET, **c**) in Northeast China (NEC).

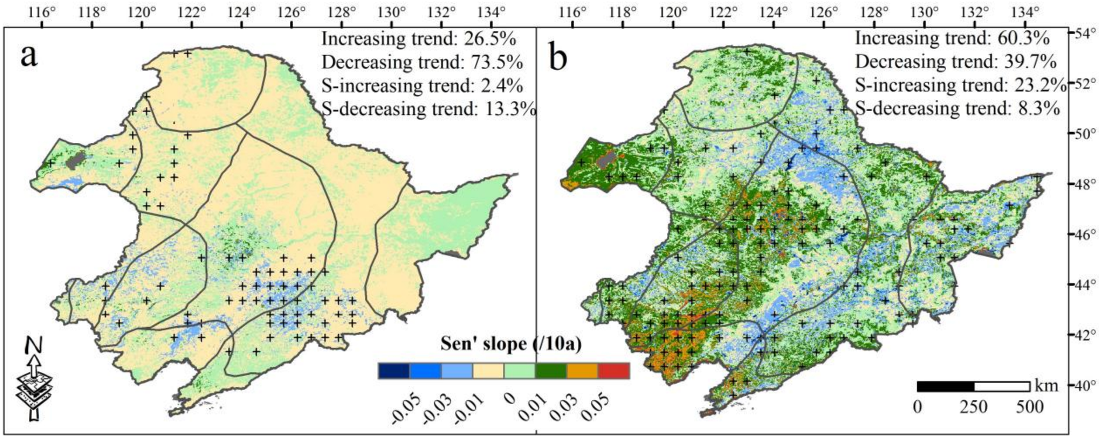

Figure S6. Spatial distribution of the trends of NDVI impacted by climate change (*$NDVI_{pre}$*, **a**) and NDVI impacted by human activities (*$NDVI_{res}$*, **b**). S- in the figure is the abbreviation for significant. Black crosses indicate significant Pearson correlations with $p < 0.05$.

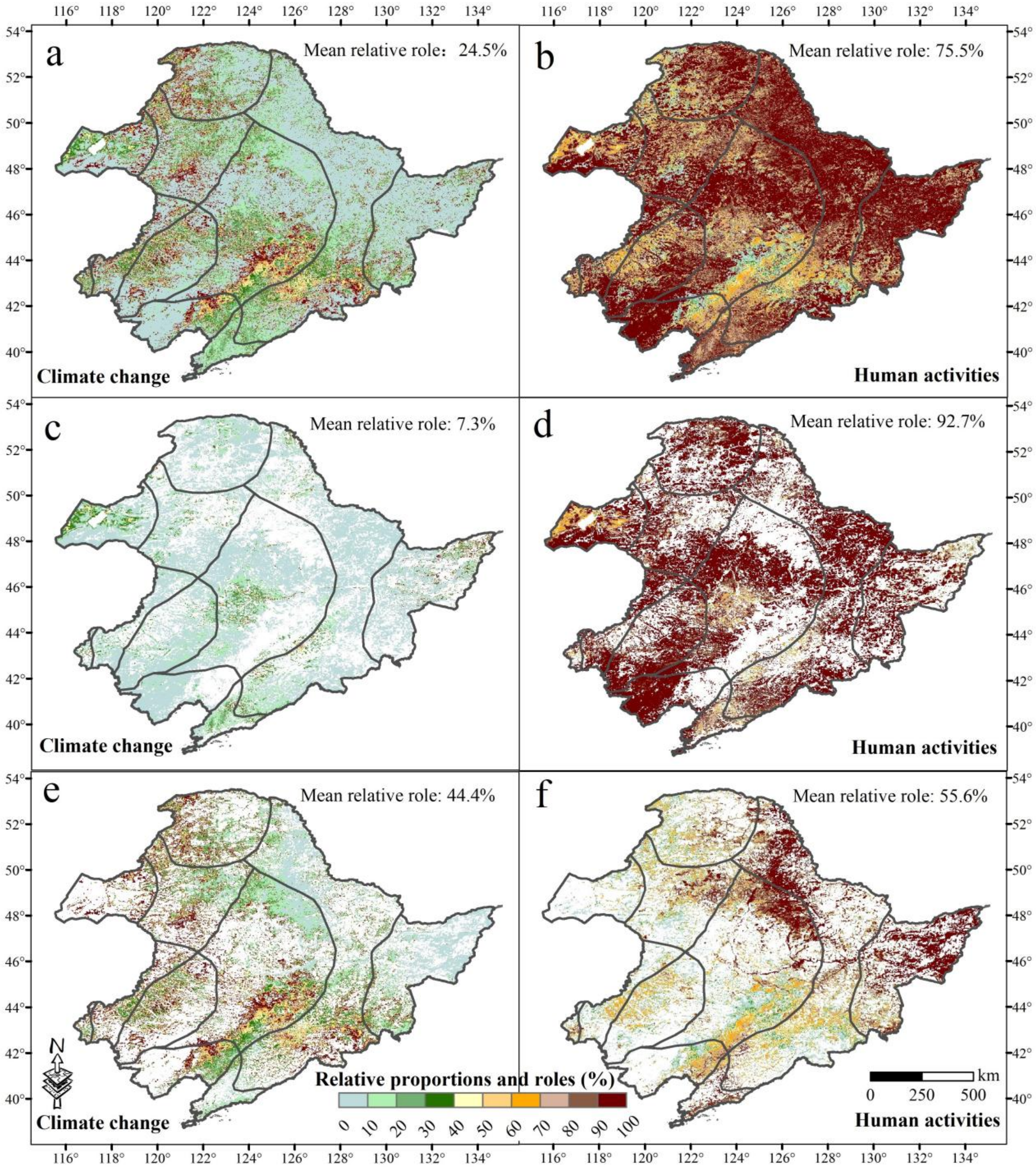

Figure S7. The spatial distribution of the relative proportions and roles of climate change and human activities in areas with observed NDVI (*NDVI$_{obs}$*) changes (**a, b**), *NDVI$_{obs}$* increasing (**c, d**), and *NDVI$_{obs}$* decreasing (**e, f**), respectively.
